# Repetitive transcranial magnetic stimulation for stimulant use disorders (STIMULUS): protocol for a multi-site, double-blind, randomized controlled trial

**DOI:** 10.1186/s13722-025-00567-w

**Published:** 2025-05-08

**Authors:** Zahraa Atoui, Donald Egan, Manish Kumar Jha, Karen Hartwell, Russell Toll, Susan Sonne, Brenda Brunner-Jackson, Geetha Subramaniam, Jenna L. McCauley, Madhukar Trivedi, Kathleen Brady

**Affiliations:** 1 Medical University of South Carolina, Charleston, SC 29425 USA; 2 UT Southwestern Medical Center, Dallas, TX USA; 3 National Institute on Drug Abuse, North Bethesda, MD USA

**Keywords:** Cocaine, Methamphetamine, Stimulant use disorder, Brain stimulation, Repetitive transcranial magnetic stimulation

## Abstract

**Background:**

Cocaine and methamphetamine use disorders (CcUD/MtUD) have serious public health, medical, and psychiatric consequences. Yet, there are no U.S. Food and Drug Administration (FDA) approved treatments available. The STIMULUS study is a multi-site trial, sponsored by the National Drug Abuse Treatment Clinical Trials Network (CTN), that aims to investigate the feasibility and preliminary efficacy of repetitive transcranial magnetic stimulation (rTMS) as a potential treatment for moderate to severe CcUD/MtUD.

**Methods:**

The study is a double-blind, sham-controlled trial seeking to recruit 160 participants with a current moderate to severe CcUD or MtUD diagnosis, randomized to receive active rTMS (10-Hz stimulation at 120% motor threshold over the left dorsolateral prefrontal cortex) or sham. Feasibility is assessed by a target of at least 20 treatment sessions administered within an 8-week period. Additionally, the study aims to evaluate the efficacy of rTMS in reducing stimulant use and craving, the impact of rTMS on mood, anxiety, sleep, and other measures, and the utility of electroencephalography as a treatment response biomarker.

**Discussion:**

Studies exploring rTMS for stimulant use disorders remain limited by small sample sizes, as well as great heterogeneity in defined study population, treatment parameters, retention in treatment, and number of sessions. In this paper, we highlight key study design decisions, such as safety, sham procedure, and schedule flexibility.

**Conclusion:**

We hope that the data collected will lay the groundwork for a robust randomized controlled trial of rTMS as a therapeutic intervention for individuals with CcUD/MtUD.

**Trial registration:**

http://www.ClinicalTrials.gov. Identifier: NCT04907357.

**Trial data set:**

https://clinicaltrials.gov/study/NCT04907357?tab=table.

**Protocol:**

Version 7.0, 11/10/2023.

**Supplementary Information:**

The online version contains supplementary material available at 10.1186/s13722-025-00567-w.

## Introduction

Cocaine and methamphetamine are psychostimulants with high addiction potential and increasing prevalence of use across the United States (U.S.), with an estimated 4.8 million and 2.5 million Americans having used cocaine and methamphetamine, respectively, in 2021 [1]. Cocaine and methamphetamine use disorders (CcUD/MtUD) substantially contribute to rising overdose deaths as well as morbidity in the form of physical and mental illnesses. From 2021 to 2022, cocaine overdose death rates in the US increased by 12.3% and accounted for one-quarter (25.5%; approximately 28,000) of all overdose deaths, whereas for methamphetamine and other psychostimulants, deaths increased by 4% and accounted for close to one-third (31.5%, approximately 39,000) of overdose deaths (~ 107,941) [2]. CcUD/MtUD are associated with a myriad of medical and psychiatric conditions (e.g., increased rates of cardiovascular, kidney, and infectious diseases, sexual risk behaviors, serious mental illness, and comorbid substance use) and have produced a significant burden for the U.S. healthcare and legal systems [3, 4]. Currently, there are no FDA-approved pharmacotherapies for the treatment of either disorder [5]. Psychological treatments exist for CcUD/MtUD, but some have modest effects with high rates of relapse [6, 7], and others, such as contingency management, remain challenging to implement. Taken together, the high disease burden combined with low rates of treatment success make CcUD/MtUD a critical target for development of novel and effective treatments.

One of the primary brain circuits implicated in CcUD/MtUD is the mesolimbic reward pathway. Cocaine and methamphetamine have been shown to increase dopamine transmission throughout the mesolimbic system, which is implicated in drug-seeking and relapse [8]. With repeated cocaine/methamphetamine exposure, the increase in dopaminergic transmission is thought to modulate the glutaminergic system in the prefrontal cortex (PFC), involved in executive function, and further contribute to drug-seeking [9, 10]. The imbalance between executive control and reward networks can result in impaired control, cravings, and drug addiction [11, 12]. This understanding of the brain networks underlying addiction has informed investigations into more targeted treatments for substance use disorder (SUDs).

Transcranial magnetic stimulation (TMS) is a non-invasive brain stimulation technique currently approved and in wide use for the treatment of major depressive disorder (MDD) and most recently (August 2020), the BrainsWay Deep TMS received FDA clearance as an aid for smoking cessation. Repetitive TMS (rTMS) refers to repetitive episodes of stimulation, which is generally the protocol for therapeutic use. rTMS targets surface brain regions, typically the prefrontal cortex, and exerts indirect effects to deeper brain circuits. High frequency rTMS (5–25 Hz) increases cortical excitability [13], while low frequency (1 Hz) application is inhibitory [14]. Consequently, specific networks involved in addiction, such as the executive network, could be engaged by brain stimulation for therapeutic benefit in individuals with SUDs [15].

Multiple studies over the past decade have investigated the safety and benefit of rTMS for individuals with stimulant use disorder (StUD) [15]. The cortical area most commonly targeted is the left dorsolateral prefrontal cortex (DLPFC) [16–19] as preliminary studies suggest that the potentiation of this circuit using high frequency stimulation counteracts the hypoactivity in the PFC that generally results from chronic addiction [20]. Studies using 10 Hz stimulation targeting the left DLPFC in men [21] and women [18] with MtUD have shown decreased craving and reduction in use. Other studies employing theta burst stimulation (TBS), a form of rTMS with shorter administration time, have also shown reduction in cue-induced cravings for methamphetamine compared to sham [22], and less days-of-use and money spent on cocaine [19]. Across different rTMS studies in SUDs, high frequency rTMS over the left DLPFC has most consistently shown evidence for positive outcomes in CcUD and MtUD [15, 23]. Despite the promise shown by this body of work, rTMS studies for StUDs remain limited by small sample sizes and great heterogeneity in treatment parameters [24]. In addition, individuals with CcUD/MtUD present with unique challenges due to their complex social, medical, and psychiatric needs. Many individuals with CcUD/MtUD struggle with unstable housing [25] and poor access to care [26]. They also present with various medical and psychiatric comorbidities [27]. These factors may significantly affect outcomes and engagement in treatment services [26] and emphasize the importance of conducting a feasibility study.

The primary objective of the protocol (STIMULUS) described in this manuscript is to determine the feasibility of rTMS for individuals with moderate to severe CcUD or MtUD. The secondary objective is to gather preliminary data regarding the efficacy of rTMS in this population. Exploratory objectives include examining the impact of rTMS on self-reported substance use, craving, mood, anxiety, sleep, treatment retention, changes in health, lifestyle, and function, and resting connectome biomarkers measured by electroencephalography (EEG).

## Study design and procedures

### Study design

**Fig. 1:**
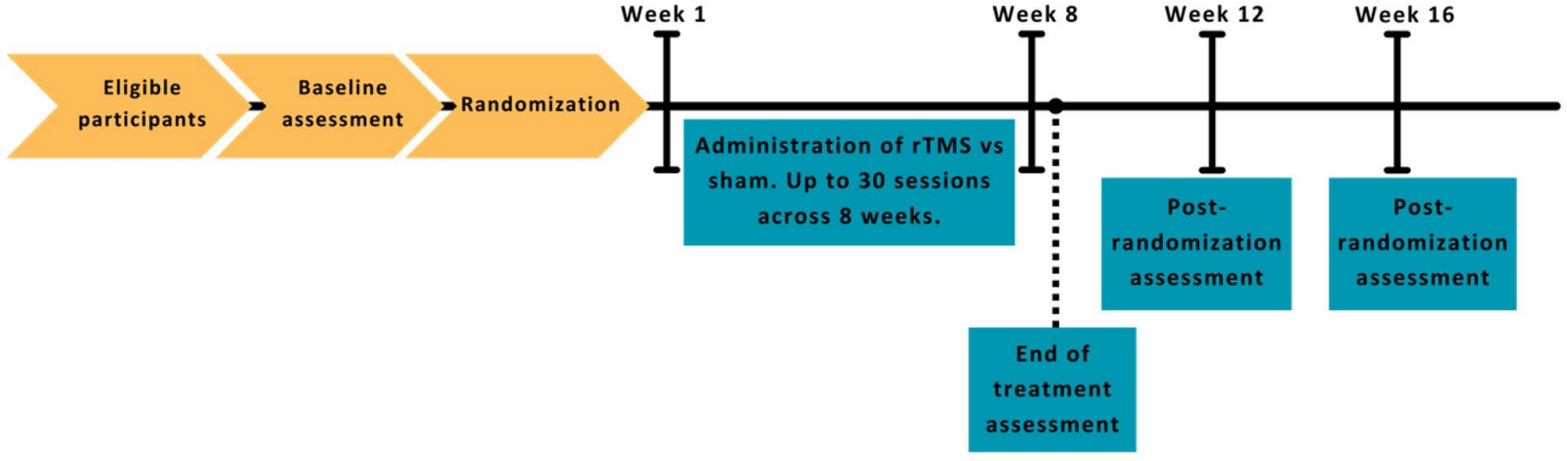
Overview of study timeline. Legend: rTMS: repetitive transcranial magnetic stimulation

The study is a randomized, double-blind trial comparing rTMS with sham treatment in reducing stimulant use among individuals with CcUD/MtUD. Potential participants are screened for eligibility and undergo an assessment process. Eligible and interested participants are randomized to receive up to 30 sessions of rTMS or sham over the course of an 8-week treatment period. Assessments are conducted at screening, weekly during treatment, end of treatment, and at 12- and 16-weeks post-randomization to determine the durability of stimulation effects (Fig. 1). Participants are compensated for participating in rTMS/sham sessions and assessments. Urine drug screens (UDS) are obtained at assessment and prior to each treatment session. The protocol is approved by the National Institute of Drug Abuse (NIDA) Center for Clinical Trials Network (CCTN) upon recommendation by the NIDA-appointed Protocol Review Board (PRB), Medical University of South Carolina (MUSC) Institutional Review Board (IRB), as well as University of Texas Southwestern (UTSW), UT Health San Antonio, and Wake Forest University IRB (CTN Protocol ID: CTN-0108; ClinicalTrials.gov ID: NCT04907357).

### Study setting

This study includes four enrolling sites which are academic medical institutions affiliated with existing CTN nodes, selected based on their experience with rTMS, as well as demonstrated history of recruitment of individuals with StUDs for research. All study sites are covered through a SMART IRB master reliance agreement with MUSC IRB as a single IRB (sIRB). Any necessary protocol amendments are submitted for sIRB approval before implementation, which in turn sends electronic notifications of approval or changes required directly to the participating sites.

### Recruitment

Participants are recruited through a variety of sources and methods including flyer posting in high traffic locations with a likelihood of being viewed by the study’s target demographic, partnerships with healthcare clinics that serve target population (e.g., infectious disease, federally qualified health centers, substance use disorder treatment facilities, community housing agencies, and other community-based non-profit organizations). Additionally, most sites have paid for forms of advertising that included social networking.

### Screening, eligibility, and randomization

The study aims to enroll 160 eligible individuals aged 18–65 years who meet criteria for a current moderate to severe CcUD or MtUD based on the Diagnostic and Statistical Manual of Mental Disorders, Fifth Edition (DSM-5). Interested individuals are prescreened either in-person or via phone (prescreen questions in Table 1). Potentially eligible individuals are scheduled for in-person screening which takes place over a minimum of 2 visits. Informed consent is obtained in writing at the outset of the first screening visit by designated study research staff prior to any study procedures being performed. If the individual maintains interest and is deemed eligible (study inclusion/exclusion criteria in Table 2/Table 3), they are randomized 1:1 to either rTMS or sham rTMS condition. Randomization is stratified based on study site, CcUD versus MtUD diagnosis, and presence of a current major depressive episode (since rTMS is FDA-approved for MDD). For this double-blinded study, the randomization is programmed into the TMS machine ensuring that neither study staff nor the participant is aware of the assignment. The randomization schedule is provided centrally through the CTN Data and Statistics Center (DSC). Unblinding is only permitted in cases of medical emergency, when knowledge of the treatment group may be necessary for clinical decision-making.

**Table 1:** Pre-screen form

	Date of assessment:	___ ___ / ___ ___ / ___ ___ ___ ___ (mm/dd/yyyy)
1.	Referral source:	☐ Flyer ☐ Public transit ad ☐ Social media ☐ Clinicaltrials.gov ☐ Word of mouth ☐ Radio ad ☐ Craigslist ☐ Other ☐ Newspaper ad ☐ TV ad ☐ Clinical referral
	a. If “Other”, specify:	_____________________________________________________________
2.	Was the participant eligible from Pre- Screen?	☐ No ☐ Yes
	a. If “No”, reason not eligible? *(select all that apply)*	☐ Less than 18 years of age or greater than 65 years of age ☐ Has not used cocaine or methamphetamine on at least 10 of the last 30 days ☐ Did not express interest in decreasing stimulant use ☐ Currently engaged in formal treatment for stimulant use disorder ☐ Currently pregnant ☐ Unwilling to use effective birth control during study ☐ Has previously received TMS in a clinical setting ☐ Has serious medical problem that would preclude safe or consistent participation in the study ☐ History of unprovoked seizure (lifetime) or any seizure in last 6 months ☐ History of brain lesion(s) and/or tumor(s) ☐ Current moderate or severe SUD, other than CcUD or MtUD ☐ Currently prescribed anticonvulsants or benzodiazepines, but not on stable dose for at least 4 weeks ☐ Suicidal or homicidal ideation ☐ Is a prisoner or in police custody ☐ Expected to be prisoner/in custody soon ☐ Metal implants or non-removable metal objects above the waist ☐ Current/lifetime history of mania or hypomania ☐ Previously randomized as a participant in the study ☐ Planned admission to a residential treatment or other formal SUD treatment program ☐ No longer interested in the study ☐ Lives too far away/transportation issues ☐ Other
	l. If “Other”, specify:	
3.	If eligible, was the participant scheduled for a screening visit?	☐ No ☐ Yes
	a. If “No”, reason not scheduled?	☐ No longer interested ☐ Other
	l. If “Other”, specify:	_____________________________________________________________
Comments: ________________________________________________________________________________________________________________________________		

*Abbreviations used: TMS: transcranial magnetic stimulation; SUD: substance use disorder; CcUD: cocaine use disorder; MtUD: methamphetamine use disorder.*

**Table 2:** Inclusion criteria and rationale

Criterion	Rationale	Assessment	Administered By
Age 18–65, inclusive.	Defines study population.	Demographics	RA/RC
Current diagnosis of moderate or severe Cocaine or Methamphetamine Use Disorder (CcUD/MtUD).	Allows for detection of rTMS versus sham effects that may not be apparent with mild CcUD or MtUD.	DSM-5 Checklist– Substance Related Disorders	RA/RC
Have used cocaine or methamphetamine on at least 10 of the last 30 days.	Emphasizes having a current CcUD or MtUD.	TLFB	RA/RC
Be willing to decrease cocaine and/or methamphetamine use, but not engaged in formal SUD treatment.	Individuals should have some motivation to attend rTMS sessions (i.e., desire to cut back or quit); however, engagement in formal treatment could confound any study treatment effects.	Treatment Status Form	RA/RC
Be able to speak English sufficiently.	To comply with ethical standards, and to understand the study procedures, risks, and benefits and provide written informed consent to participate in the study.	Consent Document and Consent Comprehension Tool	RA/RC
If prescribed benzodiazepines or anticonvulsants, must be on a stable dose for at least 4 weeks prior to consent.	These medications can affect seizure threshold and interfere with the efficacy of rTMS.	Prior and Concomitant Medications; Medical and Psychiatric History	RA/RC/MC; MC

*Abbreviations used: CcUD: cocaine use disorder; MtUD: methamphetamine use disorder; SUD: substance use disorder; rTMS: repetitive transcranial magnetic stimulation; DSM-5: Diagnostic and Statistical Manual of Mental Disorders*,* 5th Edition; TLFB: Timeline Follow-Back; RA: Research Assistant; MC: Medical Clinician.*

**Table 3:** Exclusion criteria and rationale

Criterion	Rationale	Assessment	Administered By
A DSM-5 diagnosis of moderate or severe SUD other than Tobacco Use Disorder.	Defines study population to assess effects of rTMS versus sham on CcUD or MtUD; Safety.	DSM-5 Checklist– Substance Related Disorders	RA/RC
History of a serious medical disorder that would make it unsafe to participate in the study or may prevent collection of study data.	Treatment for a serious medical disorder should take precedence over this experimental treatment for SUD.	Medical and Psychiatric History	MC
Is currently engaged in formal SUD treatment or has a planned admission to a residential treatment facility or other SUD treatment program.	To assess the effects of rTMS versus sham on CcUD or MtUD. Formal treatment for SUD would interfere with assessment of study outcomes.	Treatment Status Form	RA/RC
Documented history of unprovoked seizure (lifetime) or any seizure in the past 6 months.	Seizure is a known risk associated with rTMS; Safety.	Medical and Psychiatric History	MC
Documented history of brain lesion(s) and/or tumor(s).	The presence of a brain tumor or lesion might be a contraindication for TMS; Safety.	Medical and Psychiatric History	MC
Metal implants or non-removable metal objects above the waist.	rTMS produces strong, localized magnetic field; Safety.	Medical and Psychiatric History	MC
Current pregnancy.	While there is no available evidence to date suggesting that TMS is harmful during pregnancy, the risks of using rTMS with pregnant women are unknown.	Pregnancy and Birth Control Assessment	RA/RC/MC
History of prior TMS treatment in lifetime.	Prior TMS exposure could unblind active and sham groups.	Medical and Psychiatric History	MC
Current or lifetime manic or hypomanic episode.	Mania and hypomanic episodes may be worsened by TMS.	MINI; Medical and Psychiatric History	RA/RC; MC
Current psychotic disorder.	To keep the study population more homogeneous; participant retention.	MINI; Medical and Psychiatric History	RA/RC; MC
Current active suicidal or homicidal ideation.	Participant safety.	Medical and Psychiatric History; CHRT-SR; CHRT-CR	MC; Participant; MC
Are a prisoner or in police custody at the time of eligibility screening.	Prisoner or police custody status would preclude engagement in the study, which involves multiple in-person treatment and follow-up visits.	Screening Checklist	RA/RC
Previously randomized as a participant in the study.	Participants may only enroll in the study once.	Screening Checklist	RA/RC
Unwilling or unable to follow study procedures.	Participant safety and to allow assessment of study outcomes.	Investigator Judgment	PI

*Abbreviations used: DSM-5: Diagnostic and Statistical Manual of Mental Disorders*,* 5th Edition; SUD: substance use disorder; TMS: transcranial magnetic stimulation; rTMS: repetitive transcranial magnetic stimulation; CcUD: cocaine use disorder; MtUD: methamphetamine use disorder; MINI: Mini International Neuropsychiatric Interview; CHRT-SR: Concise Health Risk Tracking - Self Report Suicidal Behavior Evaluation; CHRT-CR: Concise Health Risk Tracking– Clinician Rated; RA: Research Assistant; MC: Medical Clinician; PI: Principal Investigator.*

### Data management

Study data is collected and entered into electronic case report forms (eCRFs) in Advantage eClinical, which is a web-based distributed data entry system. Data from actigraphy devices are uploaded and transferred securely to CTN DSC, as well as EEG data and information about Cognitive Behavioral Therapy (CBT) module usage. Each participant’s research data is identified by a unique study identification number. All data is secured and password protected. eCRFs are monitored for completeness and accuracy throughout the study by the DSC. PRB reviews the protocol before approval and periodically during study implantation and also serves as the Data and Safety Monitoring Board (DSMB). Members of this board are appointed by CCTN and are confirmed to have no competing interests.

### Treatment retention

CcUD/MtUD populations present with unique challenges to research engagement. Consequently, several processes are utilized to maintain involvement throughout the follow-up period. Research staff discuss and plan the iterative treatment schedule with patients at the outset of the study, including flexibility that accommodates participants’ other commitments within the broad intervention parameters of the study. Appointment reminders are sent to participants via their preferred contact method one week and one day prior to their follow-up. Future appointments are scheduled/confirmed with the patient at the end of each visit. Participants receive adequate compensation for initial visit, daily assessments, treatment sessions, and follow-up visits. Participants are also provided with a smart-phone device to complete daily assessments for the duration of the study or reimbursed for data plan usage if they elect to use their own device.

## Intervention

### rTMS equipment and operator training

This study uses the MagVenture MagPro^®^ X100 system equipped with a B65 active/placebo coil for double blinding. Active or sham treatment is delivered based on the orientation of the coil. Each study site designated rTMS Advanced Operator(s) specifically trained to conduct stimulation mapping, motor threshold (MT) determination, and treatment delivery. Sites may also have additional staff certified by MagVenture as TMS Operators, capable of performing treatment sessions using the MagPro^®^ system. Training includes didactics, quizzes, and passing a rigorous practicum attested by either the site PI or a designated rTMS expert at each site. Each site also has a trained medical clinician (MD, DO, PA, or NP) to assess and monitor the health and safety of participants undergoing rTMS.

### rTMS treatment dose and frequency

Stimulation mapping and determination of each participant’s MT is done at the initial rTMS treatment session. A cap is fitted on the participant’s head, stimulation mapping is then performed using the BeamF3 method, which is based on the international 10–20 system for EEG electrode placement. This method utilizes specific scalp measurements that account for individual head size and shape in order to locate the F3 position, which correlates with the left DLPFC [28]. The BeamF3 method has been shown to provide a reasonable approximation of the left DLPFC area when compared to MRI-guided neuronavigational methods [29]. The resting MT for each participant is then determined using the Parameter Estimation by Sequential Testing (PEST) algorithm, which calculates the lowest possible intensity capable of eliciting a thumb twitch in 50% of the stimulations. During treatment sessions, study staff ensure that the device is set to “Research Mode”, which will automatically allow the delivery of either active or sham treatment while maintaining the blind for both the participant and operator, and the configuration of the device to the appropriate stimulation parameters.

The treatment dose for this study is concordant with the FDA-approved stimulation dose for MDD [30] and the stimulation used in smoking cessation studies [31]. The figure-8 coil is placed over the left DLPFC. A 10-Hz stimulation at 120% MT is delivered: 75 trains for 4 s (40 pulses/train) with inter-train interval of 11 s. This provides a total of 3000 stimuli per session over 19 min. Prior to each session, participants are presented with their completed “Identify Your Triggers” worksheet, which identifies personal triggers to using substances, and asked to re-imagine a previous experience over one-minute. A set of methamphetamine or cocaine-related visual cues are then presented immediately before an active or sham stimulation is delivered. Inducing cravings before delivering rTMS is thought to enhance treatment efficacy through activating relevant brain circuits, as shown in nicotine dependence studies where adding smoking cues improved outcomes [32]. Several StUD studies adopted a similar approach, using brief drug-related cues before stimulation [18, 33]. For the current trial, a visual analog craving scale is used to measure cravings before and after treatment. A topical anesthetic (EMLA or lidocaine) is available should the participant request it. Participants may receive up to 30 sessions over an 8-week period and are encouraged to schedule 3–5 sessions per week, for a minimum of 20 total sessions. In the absence of definitive data on the optimal number of rTMS sessions for the treatment of stimulant use disorder, we decided to use 20 to 30 treatment sessions which is considered a standard acute course for the treatment of MDD [34]. However, individuals with SUDs may struggle with maintaining daily treatment attendance due to several barriers, including housing instability and lack of transportation [25, 26]. Consequently, a flexible treatment schedule is allowed to minimize dropout rate and maximize the potential for receiving at least 20 sessions in line with the study’s primary objective.

### rTMS ramping procedure

The initial study protocol included a fixed-length approach to ramping the stimulation intensity that allowed for treatment to be delivered between 100% and 120% MT for the first two sessions. Within the first 4 months of protocol implementation, it became apparent that a more flexible ramping approach would help account for individual differences in tolerability and reduce initial discomfort that may be associated with rTMS. The protocol was amended to allow for a more gradual ramping: starting at 20% of machine output (or lower if not tolerated) and titrating in increments of 2–5% until the targeted dose of 120% is achieved or the participant reports the dose is intolerable. The amended approach lengthened the ramping period from first 2 to the first 10 treatment sessions and allowed individuals who failed to reach 120% MT by the tenth session to be retained in treatment for the full study duration. If the target dose is not achieved after 10 sessions, the highest tolerable dose is used for subsequent treatments with a re-attempt to ramp every 5 sessions.

### Sham intervention

Achieving a proper sham condition that mimics the sound and feeling of active treatment is a major challenge for rTMS studies [35]. To address this, a dual active/sham coil is used to ensure that the sham administration is as similar as possible to active treatment for participants and researchers, hence maintaining the double-blind condition. This dual system delivers identical sounds in both conditions. In the sham group, the magnetic field is delivered away from the brain, which does not interfere with the targeted brain circuit.

### Concurrent CBT modules

Given the experimental nature of the study and to isolate the effects of rTMS, participants are not engaged in formal SUD treatment at the time of enrollment, and it is not provided throughout the study. Nonetheless, participants are asked to participate in a CBT intervention specific for SUDs. This intervention aims to provide standardized and evidence-based care as it is our ethical obligation to provide treatment for all participants. The CBT is delivered using the CBT modules offered by DynamiCare Health via a mobile application platform [36]. If needed, phones are provided to ensure access. Twenty self-guided therapy-modules deliver evidence-based CBT and motivational interviewing therapeutic content (e.g. Healthy Coping Skills, Relapse Prevention Plan and Tips, Mindfulness). Participants are asked to complete 2–3 modules per week during the treatment phase. Modules are brief, available anytime, contain both didactic and interactive content, and may be completed in single/multiple sessions and in any order (see Additional File 1 for CBT session topics). Participation is tracked by research staff but is not incentivized. Use of the modules may influence the study outcome regarding stimulant use; however, the use of CBT modules is exploratory and not a part of the final analysis.

## Outcomes and assessments

### Primary outcome

The primary objective is to determine the feasibility of up to 30 sessions of active rTMS over the left DLPFC versus up to 30 sessions of sham rTMS in individuals with CcUD or MtUD. The feasibility outcome is the percentage of participants who receive at least 20 sessions over the 8-week treatment period. Treatment session attendance and completion is tracked over the course of the study. In addition to the primary feasibility outcome, data on the pattern of rTMS sessions and dose delivered will be analyzed and reported.

### Secondary outcome

The secondary objective is to examine the effect size of up to 30 sessions of rTMS over the left DLPFC versus up to 30 sessions of sham rTMS on cocaine or methamphetamine use in individuals with CcUD/MtUD. The efficacy outcome is determined using the last UDS in each treatment week. The percent of negative UDS from these 8 samples is presented as a continuous measure. A negative screen for participants in the CcUD group would be a UDS absent of cocaine; a negative screen for participants in the MtUD group would be a UDS absent of methamphetamine.

### Exploratory outcomes

Exploratory objectives include the impact of rTMS on self-reported substance use, craving, mood, anxiety, sleep monitored via actigraphy, resting connectome profile via EEG, treatment retention, and changes in health, lifestyle, and function. The Actigraph GT9X Link is an FDA-approved wristband which monitors sleep latency, duration, and waking intervals. Sleep data are crucial for understanding the baseline state of participants and how it may influence their response to rTMS, particularly given that sleep disturbances are common in SUDs and can affect cravings and relapse rates [37]. EEG is another impactful measure in the study. Baseline and mid-treatment (week 4) resting EEG is acquired, allowing for computation of longitudinal changes in functional network connectivity (detailed methodology in Additional File 2), which could help identify treatment response biomarkers. To augment data collection, daily remote assessments/surveys are administered to participants on their mobile devices from day 1 of treatment through week 16 follow-up. The survey asks participants to answer whether they used their primary reported substance (Yes/No) and to rate, using a Visual Analog Scale, their craving, ability to resist use of primary substance, overall mood, and sleep quality. Table 4 presents assessments administered across this study, identifying the purpose and/or core construct assessed by each measure. Table 5 provides a schedule for administration of these assessments.

**Table 4:** Study assessments and brief description

Assessment	Description	Administered By
Locator Form	Collects the participant’s current address, email address, and phone numbers to assist in finding participants during study participation.	RA/RC
Phen-X Toolkit Core Tier 1	Includes measures for demographics, BMI, HIV risk and status, and other substance use measures such as age of onset.	RA/RC
Protocol Satisfaction Form	Provides information on the participant’s satisfaction with the study and study procedures.	Participant
Physical Examination	Assesses whether individuals are medically stable for study inclusion.	MC
Medical and Psychiatric History	Covers past and present health conditions to help determine eligibility and to provide baseline information.	MC
Vital Signs	Obtaining valid measures of weight, blood pressure, and pulse.	RA/RC/MC
Prior and Concomitant Medications	Documents all medications taken by the participant for the 30 days prior to and during screening/baseline and during the active study for safety purposes.	RA/RC/MC
Penetration of Blind	Participants and primary study personnel are asked whether they think the participant is receiving rTMS or sham.	Participant, RA/RC, MC
EEG	Assesses resting connectome profile as a potential treatment response biomarker.	RA/RC or EEG technician
MINI 7.0.2	Semi-structured interview designed to ascertain a current, past, or lifetime history of the major Axis I psychiatric disorders in DSM-5.	RA/RC
HADS	Brief, validated instrument that screens for both depression and anxiety.	Participant
PSQI	Brief, validated instrument that measures sleep quality.	Participant
CHRT-SR	16-item self-report assessment of suicidality and related thoughts and behaviors.	Participant
CHRT-CR	Performed if a participant screens positive on the CHRT-SR.	MC
TLFB	Used to elicit the participant’s self-reported use, quantity, and route of administration of substances of abuse.	RA/RC
Caffeine Intake Assessment	Assesses and quantifies use of caffeinated beverages.	RA/RC
DSM-5 SUD Symptom Checklist	Semi-structured, interviewer-administered instrument that provides current diagnoses for substance use disorders based on DSM-5 diagnostic criteria.	RA/RC
NIDA Cannabis Use Assessment	Assesses recreational and medical cannabis use frequency over the past 12 months, including reasons for use, method of administration, and perceived harm or benefit associated with use.	Participant
Fagerström Test for Nicotine Dependence	Assesses cigarette use and nicotine dependence.	Participant
Cue Craving Assessment	Assesses participant’s craving level after being exposed to cocaine/methamphetamine cues and after having the rTMS/sham intervention.	RA/RC
Actigraphy	Collects participants’ daily sleep data, including sleep latency, duration, and waking intervals.	RA/RC
Daily Assessments	Assesses daily substance use, cravings, stress, ability to resist use, mood, and sleep quality. The survey is delivered to the participant’s mobile device via a HIPAA-compliant application.	Participant
Urine Drug Screen	To assess several secondary outcome measures.	RA/RC
Urine Pregnancy Test	To determine eligibility for female participants.	RA/RC

*Abbreviations used: Phen-X: Phenotypes and Exposures; EEG: Electroencephalogram; MINI 7.0.2: Mini International Neuropsychiatric Interview Plus; HADS: Hospital Anxiety and Depression Scale; PSQI: Pittsburgh Sleep Quality Index; CHRT-SR: Concise Health Risk Tracking—Self Report Suicidal Behavior Evaluation; CHRT-CR: Concise Health Risk Tracking– Clinician Rated; TLFB: Timeline Follow-Back; DSM-5: Diagnostic and Statistical Manual of Mental Disorders*,* 5th Edition; SUD: substance use disorder; NIDA: National Institute on Drug Abuse; BMI: body mass index; HIV: Human Immunodeficiency Virus; rTMS: repetitive transcranial magnetic stimulation; HIPAA: Health Insurance Portability and Accountability Act; RA: Research Assistant; RC: Research Coordinator; MC: Medical Clinician.*

**Table 5:** Study assessments timeline

	SC	Double-Blind Treatment Phase								Post	FU	FU
Week	0	1	2	3	4	5	6	7	8	EOT	12	16
**Administrative Forms**												
Informed Consent												
Inclusion/Exclusion Review	X											
Locator Form and Updates*	X	X	X	X	X	X	X	X	X	X	X	
Daily TMS Treatment Log		X	X	X	X	X	X	X	X			
End of Treatment Form										X		
Study Completion												X
**General Assessments**												
Phen-X Toolkit Core Tier 1	X											
Phen-X Toolkit Quality of Life	X									X	X	X
Study Demographics Form*	X											
Treatment/Study Satisfaction Form										X		X
**Medical Assessments**												
Physical Exam	X											
Medical and Psychiatric History	X											
Weight, Blood Pressure, and Pulse	X	X				X				X	X	X
Adverse Events, Serious Adverse Events, and Medical Review	X	X	X	X	X	X	X	X	X	X	X	X
Prior/Concomitant Meds	X	X	X	X	X	X	X	X	X	X	X	X
Penetration of Blind Assessment						X				X		X
EEG	X				X							
**Psychological Assessments**												
MINI 7.0.2*	X											
HADS	X				X				X	X	X	X
Pittsburgh Sleep Quality Index	X				X				X		X	X
CHRT-SR Suicidal Behavior Eval	X	X	X	X	X	X	X	X	X	X	X	X
**Substance Use Self Report**												
TLFB/Substance Use Diary	X	X	X	X	X	X	X	X	X	X	X	X
Caffeine Consumption Assessment	X	X	X	X	X	X	X	X	X			
DSM-5 SUD Symptom Checklist*	X											
NIDA Marijuana Use Assessment	X											
Fagerström Test for Nicotine Dependence	X				X					X	X	X
**Daily Assessments**												
Visual Analog Craving Scale	X	X	X	X	X	X	X	X	X	X	X	X
Report of Methamphetamine and/or Cocaine Use	X	X	X	X	X	X	X	X	X	X	X	X
Report of Nicotine Use	X	X	X	X	X	X	X	X	X	X	X	X
Mood	X	X	X	X	X	X	X	X	X	X	X	X
Sleep	X	X	X	X	X	X	X	X	X	X	X	X
Actigraphy	X	X	X	X	X	X	X	X	X			
**Lab Testing**												
UDS (dipstick)*	X	X	X	X	X	X	X	X	X	X	X	X
Urine Pregnancy Test*	X				X				X			

* During screening visit,* it is recommended to start with these assessments after consent is obtained to minimize the workload for screen failures.*

*Abbreviations used: SC: Screening; EOT: End of treatment; FU: Follow up; TMS: Transcranial magnetic stimulation; Phen-X: Phenotypes and Exposures; EEG: Electroencephalogram; MINI 7.0.2: Mini International Neuropsychiatric Interview Plus; HADS: Hospital Anxiety and Depression Scale; CHRT-SR: Concise Health Risk Tracking—Self Report; TLFB: Timeline Follow-Back; DSM-5: Diagnostic and Statistical Manual of Mental Disorders*,* 5th Edition; SUD: Substance use disorder; NIDA: National Institute on Drug Abuse; UDS: Urine drug screen.*

### Safety

The study uses stimulation parameters that have been widely used with established safety in treatment of MDD and smoking cessation. In addition, several studies have shown that rTMS is similarly tolerable and safe for individuals with SUDs [16]. A thorough medical assessment is performed by the medical clinician, including medical and psychiatric history, physical exam, and collection of vital signs prior to initiation of rTMS to identify any potential risks, such as a history of seizures. Nonetheless, a comprehensive process is also in place to identify, address, and report safety events. Common, and thus anticipated, adverse events (AEs) related to the use of rTMS include pain or discomfort at stimulation site or head/neck during treatment, and post-treatment headache. Study staff assess for AEs and serious AEs (SAEs) at every visit. All reported events are reviewed by the medical clinician and submitted to the CTN Clinical Coordinating Center for additional review by a NIDA-assigned Medical Monitor. All AEs/SAEs are followed until resolution, or up to 30 days after last study visit. The DSMB monitors the progress of the study and has access to all adverse events to determine whether the study should be temporarily suspended or terminated.

To ensure safety and tolerability of rTMS, and to minimize risk of seizure, resting MT is retested every 10 treatment sessions, or sooner at the discretion of the study medical clinician should any concern for a lowered seizure threshold arise. Certain events may trigger an unscheduled reassessment of MT, such as a newly positive UDS for illicit drugs, including cocaine or methamphetamine, after previously being negative, or if the participant describes an increase in drug use or caffeine use since the previous treatment. A breathalyzer assessment is done prior to each session. rTMS treatment is not delivered if patient is acutely intoxicated or withdrawing. Additionally, prior and concomitant medications are monitored throughout the study to ensure these do not elevate seizure risk associated with study participation.

Given the increased risk and vulnerability of the study population, a clinical deterioration “rescue” plan is in place to address clinical decompensation. Concise Health Risk Tracking—Self Report (CHRT-SR) is used to evaluate suicidal behavior at baseline, weekly during the treatment phase, and at follow-up visits. Study staff review the results prior to concluding the visit and alert the medical clinician should any concerns arise.

## Statistical analysis plan

### Primary outcome analysis

The primary outcome is feasibility of rTMS as a treatment for StUD, measured by the percentage of participants who receive 20 or more sessions (either rTMS or sham). Participants who do not complete treatment are included in the intent to treat (ITT) analysis of the primary outcome. At the end of the study, the results are summarized by the estimated percentage and a 95% confidence interval using the Wilson method.

### Secondary outcome analysis

The secondary objective is to examine the preliminary efficacy of rTMS treatment versus sham. This is analyzed using a generalized linear mixed model approach to adjust for correlation between UDS from the same participant. The percent of negative UDS from the last day of each treatment week is presented as a continuous measure. A negative drug screen for participants in the CcUD group would be a UDS absent of cocaine, while for MtUD group it would be a UDS absent of methamphetamine. The model includes fixed effects for treatment, major depressive episode, treatment week, site, and a random effect for participant. The treatment effect will be measured by an odds ratio with an associated 95% confidence interval.

### Exploratory outcomes analyses

Measures assessing exploratory outcomes are summarized and analyzed as appropriate for the outcome type. Fisher exact tests and/or logistic regression models are used for binary outcomes, while non-parametric tests of mean/median differences and other types of regression models for ordinal or continuous outcomes.

### Sample size estimate and effect size estimates

A total of approximately 160 individuals with moderate to severe CcUD (*N* = 80) and MtUD (*N* = 80), who have an interest in cutting down or stopping their use, are recruited across 4 study sites. This sample size provides adequately sized 95% confidence intervals for the primary outcome and greater than 80% power to detect medium-sized treatment effect, measured as odds ratios, of greater than 4 with a two-sided hypothesis test and significance level of 0.05 for the secondary (efficacy) outcome.

## Study design decisions

### Safety

A key consideration during screening candidates for study eligibility is limiting inclusion to participants with moderate to severe CcUD or MtUD. Aside from nicotine, participants with an additional DSM-5 diagnosis of a moderate to severe SUD are excluded from the study. Focusing on CcUD/MtUD allows for a better understanding of the specific effects of rTMS on these stimulants by avoiding any confounding variables that could be introduced through other substances. This is consistent with most studies exploring the effect of rTMS on CcUD/MtUD, where only subjects with moderate to severe SUD of respective substance are included [33, 38]. With the increasing rates of polysubstance use [39], once feasibility is established, rTMS use could be expanded to other comorbid SUDs. Should future studies include individuals with moderate-severe use disorder for substances in addition to cocaine and methamphetamine, additional safety considerations must be evaluated. The concomitant use or withdrawal from multiple substances, such as alcohol and benzodiazepines, can lower the seizure threshold and pose a potential seizure risk [40].

A second key safety consideration is the presence of non-removable ferromagnetic devices or implants. The presence of metallic objects close to the coil, such as cochlear implants [40], is a well-documented contraindication for rTMS as it could induce potential heating or displacement of the metal [41]. Ample previous work with rTMS excludes individuals with metal objects above the neck [24, 38]. However, for this study, the decision was made to conservatively exclude individuals with metal objects not only around the coil area but also above the waist given both the novelty of rTMS use in this population as well as the potential additional risks associated with stimulant drug use. This approach is primarily related to safety, as the study is an early-stage clinical trial.

### Sham procedure

The discomfort produced by the rTMS-related muscle twitching could alter the participant’s experience and ratings on subjective measures, such as craving [42]. A strong sham condition mimics the noise, muscle twitching, and potential discomfort of the active treatment. The STIMULUS study utilizes an active/placebo coil that allows for double blinding, hence providing an identical treatment setting and environment. The study also uses small electrodes attached to the scalp area adjacent to the coil for all participants. These electrodes deliver a mild electrical stimulation during sham to mimic the tactile sensation/twitching generally produced by the active treatment. The addition of electrodes considerably controls for placebo effects [43]. Even with the application of gold-standard rTMS sham procedures [44], this study also assesses both participants and study staff for penetration of blind via self-report assessments completed periodically throughout the study.

### rTMS treatment schedule

The primary objective of this trial is determining the feasibility of rTMS, therefore, considerable consideration was given to providing the greatest degree of flexibility with respect to session scheduling and initial rTMS dose ramping procedure to increase tolerability of the procedure and promote retention. In smoking cessation rTMS studies, the standard treatment consisted of daily sessions, 5 days per week, for 3 weeks, followed by weekly sessions for an additional 3 weeks [31]. For this trial, participants are provided with the flexibility to schedule up to two sessions a day (with at least one hour in between sessions) and work with study coordinators to tailor their schedule with a goal target of 2–3 sessions per week. This decision was primarily driven to optimize data collection from a traditionally difficult-to-retain patient population [45, 46], as well as COVID-19 restrictions during the earlier stages of the trial. This data, once gathered, will provide valuable insights for future studies seeking to refine scheduling flexibility and tolerability of rTMS treatments tailored to CcUD/MtUD patients.

## Discussion

The prevalence and deleterious public health impact of CcUD and MtUD are on the rise in the U.S. which poses significant medical and psychiatric challenges. Despite a growing need, there is a notable lack of effective interventions, and no FDA-approved treatment. One area of interest for treatment of SUDs is rTMS, which has been FDA-approved for smoking cessation and depression.

The current STIMULUS study is the first double-blind, multi-site, study to evaluate the feasibility of rTMS as a treatment for CcUD and MtUD. The trial incorporates specific design decisions aimed at enhancing participant retention, treatment tolerability, and the generation of informative treatment feasibility data. This study will establish a solid foundation for future research endeavors focused on the impact of rTMS as a therapeutic intervention for individuals with CcUD and MtUD, including informing future research on optimal stimulation parameters, treatment frequency and scheduling that, if promising, will progress rTMS treatment towards FDA indication for treatment of StUDs.

## Electronic supplementary material

Below is the link to the electronic supplementary material.

Supplementary Material 1

Supplementary Material 2

## Data Availability

No datasets were generated or analysed during the current study.

## Abbreviations

FDA: U.S. Food and Drug Administration
CCTN: Center for Clinical Trials Network
NIDA: National Institute of Drug Abuse
MUSC: Medical University of South Carolina
UTSW: University of Texas Southwestern
IRB: Institutional Review Board
DSC: Data and Statistics Center
DSM-5: Diagnostic and Statistical Manual of Mental Disorders, Fifth Edition
rTMS: Repetitive Transcranial Magnetic Stimulation
eCRF: Electronic Case Report Form
MT: Motor Threshold
PEST: Parameter Estimation by Sequential Testing
CcUD: Cocaine Use Disorder
MtUD: Methamphetamine Use Disorder
StUD: Stimulant Use Disorder
SUD: Substance Use Disorder
PFC: Prefrontal Cortex
DLPFC: Dorsolateral Prefrontal Cortex
MDD: Major Depressive Disorder
EEG: Electroencephalography
UDS: Urine Drug Screen
CBT: Cognitive Behavioral Therapy
AE: Adverse Event
SAE: Serious Adverse Event
DSMB: Data and Safety Monitoring Board
CHRT-SR: Concise Health Risk Tracking—Self Report
