# Supplementary material for: Repetitive transcranial magnetic stimulation for stimulant use disorders (STIMULUS): protocol for a multi-site, double-blind, randomized controlled trial
Source: Addict Sci Clin Pract. 2025 May 8;20:40. doi: 10.1186/s13722-025-00567-w (PMC12060337; doi:10.1186/s13722-025-00567-w)
Supplement: Supplementary file 1 — Supplementary Material 1 [file 13722_2025_567_MOESM1_ESM.docx]

**Additional File 1.** Cognitive Behavioral Therapy (CBT) Session Topics

| **Module Title** | **Content Summary** |
| --- | --- |
| Hijacking the Brain: Disease of Addiction | Signs of addiction: loss of control and overview of disease of addiction |
| Triggers (learning about triggers): Part 1 | Identifying personal triggers and reaction (positive and negative) to them |
| Triggers (practice scenarios): Part 2 | Practice 3 scenarios identifying triggers, and subsequent reaction/behavior |
| Healthy Coping Skills: Part 1 | Planning for encountering triggers and introduction to healthy coping options |
| Healthy Coping Skills: Part 2 | Avoiding triggers and managing/coping with unavoidable triggers |
| Justification | Identification of own justifications for substance use |
| Relapse Prevention Plan: Part 1 | Planning for triggers, implementing healthy coping plan, attaining sober support |
| Relapse Prevention Plan: Part 2 | Implementing a healthy coping plan for triggers |
| Relapse Prevention Plan: Part 3 | Identifying sober support and putting relapse prevention plan into practice |
| Relapse Prevention Tips: Part 1 | Avoiding relapse: Skills training for HALT, urge surfing, and setting healthy boundaries |
| Relapse Prevention Tips: Part 2 | Reviewing HALT, urge surfing, and boundary setting |
| Building Discrepancy – Decision Making Skills | Focus on evaluating & responding vs. reacting; Differentiating between healthy vs. unhealthy coping |
| Cognitive Behavioral Model: Part 1 | Introducing tripartite model and evaluating thoughts for cognitive distortions |
| Cognitive Behavioral Model: Part 2 | Reviewing CBT model and introducing reframing |
| Rational vs. Irrational Thoughts | Identifying irrational thoughts based in fear rather than fact |
| Self-Care Assessment | Building a healthy lifestyle via activities that promote physical, mental & emotional heath |
| Dealing with Stressors | Defining stress and identifying warning signs of chronic stress (e.g., fatigue) |
| Mindfulness | Introducing core mindfulness components of awareness and acceptance; Mindfulness practice exercise |
| Grounding Techniques | Teaching techniques including: (1) Refocusing on present moment during uncomfortable feelings, distressing memories, triggers, and (2) 5-4-3-2-1 exercise and practice |
| Using Your Strengths | Identifying personal strengths and their role in achieving personal goals |
