# Supplementary material for: Repetitive transcranial magnetic stimulation for stimulant use disorders (STIMULUS): protocol for a multi-site, double-blind, randomized controlled trial
Source: Addict Sci Clin Pract. 2025 May 8;20:40. doi: 10.1186/s13722-025-00567-w (PMC12060337; doi:10.1186/s13722-025-00567-w)
Supplement: Supplementary file 2 — Supplementary Material 2 [file 13722_2025_567_MOESM2_ESM.docx]

**Acquiring and Analyzing EEG Data**

Orthogonalized Power Envelope Connectivity

At the MUSC site, EEG data were acquired using a Compumedics Neuroscan Neuvo amplifier sampling at 1000 Hz and an interface-modified MagstimEGI HydroCel GSN 130 64 channel geodesic montage cap. At the UTSW site EEG data were acquired using a MagstimEGI GES 400 system comprised of a Net Amps 400 amplifier sampling at 1000 Hz and a HydroCel GSN 130 256 channel geodesic montage cap.

EEG data were preprocessed by high-pass filtering at 1 Hz and low-pass filtering at 45 Hz. This cutoff selects for the majority of connectivity features and mitigates the artifactual contributions of muscle activations and electrical line noise.^1^ The data were then downsampled to 250 Hz and bad channels, identified algorithmically, were interpolated using the spherical method. Paroxysmal segments were excised. Full-rank independent components analysis identified artifactual components for subtraction. Each preprocessing step was executed in MATLAB (release R2022b; MathWorks, Inc., Natick, Mass.) and its output was inspected by a human expert for quality assurance.

Source localization to the tessellated cortical surface was computed using the Brainstorm toolbox.^2^ A 3-layer symmetric boundary element model of the Montreal Neurological Institute (MNI) template calculated using the OpenMEEG plugin produced rotating dipoles at 5000 vertices at the cortical surface.^3^ The lead-field matrix was obtained by projecting the standard electrode montage appropriate for the EEG cap. An unconstrained imaging kernel mapping the channel-space EEG to source-space current density was calculated using the minimum norm estimation method with depth weighting and regularization.

EEG data were then downsampled to 100 Hz and convolved with a complex-valued Morlet wavelet of ±3-temporal standard deviations and bandwidth 4 Hz at center frequencies of 6, 10, and 14 Hz to obtain the theta, alpha, and low beta frequency bands, respectively. In each frequency band the unconstrained imaging kernel was reduced from its 3 orthogonal axes to a single principal direction by primary components analysis of the real component of the analytic signal (band-passed complex EEG). This reduced imaging kernel multiplied with the channel-space analytic signal produces the source-space analytic signal.

Plain power envelopes were obtained from these source-space data by multiplication with its complex conjugate. An orthogonalization procedure was then applied to the plain power envelopes.^4^ Vertex-wise global connectivity between plain and orthogonalized power envelopes was computed in successive, half-overlapping windows one order of magnitude larger than the Morlet wavelet duration (approximately 5 seconds). The median connectivity value across windows was used as the final representative connectivity measure producing 5000 vertices x 5000 vertices connectivity matrices for each subject-time point-frequency band combination.

These data were compared to global connectivity measures found in separate studies for quality assurance.^1,5^ Connectivity measures were recomputed using a narrow bandwidth of 1 Hz at center frequencies 2-28 Hz to produce frequency-connectivity curves at 3 landmark early sensory areas (visual, auditory, and somatosensory) to verify congruence with expected sensory connectivity curves.

Network connectivity inferences require a mapping of constituent vertices to their respective networks. Yeo’s seminal 2011 study of 1000 healthy control subjects is a standard of fMRI atlases.^6^ The 2018 Schaefer parcellations of Yeo’s atlas has further advanced this work.^7^ We applied a Schaefer parcellation (100 parcels) to the cortical tessellation for use in EEG. The 100-parcel atlas is ideal as the size of ROIs are consistent with the spatial resolution of high-density EEG. These parcels were used to classify the network membership of each vertex in the connectivity matrix.

1. Hipp, J. F., Hawellek, D. J., Corbetta, M., Siegel, M. & Engel, A. K. Large-scale cortical correlation structure of spontaneous oscillatory activity. *Nat. Neurosci.* **15**, 884–890 (2012).

2. Tadel, F., Baillet, S., Mosher, J. C., Pantazis, D. & Leahy, R. M. Brainstorm: a user-friendly application for MEG/EEG analysis. *Comput. Intell. Neurosci.* **2011**, 879716 (2011).

3. Gramfort, A., Papadopoulo, T., Olivi, E. & Clerc, M. OpenMEEG: opensource software for quasistatic bioelectromagnetics. *Biomed. Eng. Online* **9**, 45 (2010).

4. Toll, R. T. *et al.* An Electroencephalography Connectomic Profile of Posttraumatic Stress Disorder. *Am. J. Psychiatry* **177**, 233–243 (2020).

5. Hipp, J. F. & Siegel, M. BOLD fMRI Correlation Reflects Frequency-Specific Neuronal Correlation. *Curr. Biol.* **25**, 1368–1374 (2015).

6. Yeo, B. T., Krienen, F. M., Chee, M. W. & Buckner, R. L. Estimates of Segregation and Overlap of Functional Connectivity Networks in the Human Cerebral Cortex. *NeuroImage* **88**, 212–227 (2014).

7. Schaefer, A. *et al.* Local-Global Parcellation of the Human Cerebral Cortex from Intrinsic Functional Connectivity MRI. *Cereb. Cortex N. Y. NY* **28**, 3095–3114 (2018).

MATLAB Code:

function [Rplain,Rortho] = orthogonalize(K,BPCD)

% This function calculates the orthogonalized connectivity matrix (Rortho)

% as well as the plain (non-orthogonalized) connectivity matrix (Rplain)

% given a spatial imaging kernel (K) and band-passed complex EEG data (BPCD)

%

% K is a matrix obtained from an inverse solution method such as minimum norm

% estimation using the Brainstorm toolbox. It has dimensionality v vertices x n

% channels.

%

% BPCD is a complex matrix of EEG data that has been band-passed at a certain

% center frequency and bandwidth. For example, EEG data band-passed with center

% frequency 10 Hz and bandwidth 4 Hz to contain the alpha frequency band. This

% data is then made into an analytic signal via the Hilbert transform or using

% Morlet wavelets, etc. It has dimensionality n channels x t time points.

% 1. Input validation.

% 1.1. K and BPCD are required inputs

narginchk(2,2)

% 1.2. K and BPCD must be matrices.

assert(ismatrix(K),'K must be a matrix (vertices x channels).')

assert(ismatrix(BPCD),'BPCD must be a matrix (channels x time points).')

% 1.3. BPCD must be complex-valued.

assert(~isreal(BPCD),'BPCD must be a complex-valued matrix.')

% 1.4. K and BPCD must not have any non-finite values (NaNs).

assert(all(isfinite(K(:))),'Non-finite values detected in K.')

assert(all(isfinite(real(BPCD(:)))),'Non-finite values detected in BPCD.')

assert(all(isfinite(imag(BPCD(:)))),'Non-finite values detected in BPCD.')

% 1.5. Issue a warning if the imaginary component of BPCD is all zero or near

% zero values.

if nansum(nansum(round(imag(BPCD),3))) == 0

warning('The imaginary component of BPCD appears to contain all zero values, check that your band-passed complex EEG data is correct.')

end

% 2. Calculate source space (SS). Dimensionality is v vertices x t time points.

% Values are computed using single precision as the memory and computation time

% requirements increase substantially when using double precision with

% negligible gain in accuracy.

SS = single(K * BPCD);

% 3. Initialize the directional connectivity matrix (RorthoAB). Dimensionality

% is a square matrix of v vertices x v vertices.

RorthoAB = single(NaN(size(K,1),size(K,1)));

% 4. Determine if a capable GPU is present to perform the orthogonalization.

toolboxInfo = ver;

useGPU = false;

% The Parallel Computing Toolbox is required to use GPU functions.

if any(strcmp({toolboxInfo.Name},'Parallel Computing Toolbox'))

if gpuDeviceCount > 0

gpu = gpuDevice(1);

SSinfo = whos('SS');

RorthoABinfo = whos('RorthoAB');

% Check to make sure the detected GPU has enough available memory.

if gpu.AvailableMemory >= 2 * SSinfo.bytes + RorthoABinfo.bytes

useGPU = true;

else

warning('A GPU was detected, but it does not have sufficient memory. Using the CPU to perform the orthogonalization. This will take substantially longer computation time (possibly an hour or more).')

end

else

warning('No GPU was detected, using the CPU to perform the orthogonalization. This will take substantially longer computation time (possibly an hour or more).')

end

else

warning('No Parallel Computing Toolbox was detected (required to use GPUs). Using the CPU to perform the orthogonalization. This will take substantially longer computation time (possibly an hour or more).')

end

% 5. Calculate the plain power envelopes (PEplain) by multiplying SS with its

% conjugate. Dimensionality is v vertices x t time points.

PEplain = SS .* conj(SS);

% 6. Calculate the plain connectivity matrix (Rplain) by computing the

% vertex-wise correlations of the natural logarithm of the plain power

% envelopes (PEplain). An extremely small value (tol) is added to the power envelopes

% to prevent the natural logarithm resulting in a non-finite value (i.e., to prevent

% log(0) = -Inf). Dimensionality is a square matrix of v vertices x v vertices.

tol = realmin('single');

PEplain = log(PEplain + tol);

Rplain = corr(PEplain',PEplain');

% 7. Calculate the directional orthogonalized connectivity matrix (RorthoAB).

% Here, AB refers to the fact that orthogonalization is a directional operation.

% That is, vertex A orthogonalized with respect to vertex B is not the same as

% vertex B orthogonalized with respect to vertex A. This is accomplished by

% orthogonalizing SS with respect to one vertex per iteration, calculating

% the resulting orthogonalized power envelopes (PEortho) and the power envelope

% of the seed vertex (PEseed), and then computing the correlation of the natural

% logarithms of these power envelopes to obtain a measure of connectivity. As

% with the correlation of the natural logarithm of the plain power

% envelopes, an extremely small value (tol) is added to prevent non-finite values.

if useGPU

SS = gpuArray(SS);

RorthoAB = gpuArray(RorthoAB);

tol = gpuArray(tol);

end

% Display a waitbar to indicate progress.

waitbarHandle = waitbar(0,'Initializing','Name','Orthogonalized connectivity');

for iVertex=1:size(SS,1)

% Seed is the vertex that SS is being orthogonalized with respect to.

% Dimensionality is 1 x t time points.

seed = SS(iVertex,:);

% Calculate PEseed by multiplication with its conjugate.

% Dimensionality is 1 x t time points.

PEseed = seed .* conj(seed);

% Calculate PEortho by applying Hipp's equation.

% Dimensionality is v vertices x t time points.

PEortho = imag(SS .* ( conj(seed) ./ abs(seed) )).^2;

% Calculate the natural logarithm of the power envelopes to make them more normal

% and add tol to prevent non-finite values.

PEseed = log(PEseed + tol);

PEortho = log(PEortho + tol);

% Calculate the correlation between the natural logarithm of PEseed

% and the natural logarithm of PEortho. This produces a column vector

% of v correlation coefficients per iteration. The MATLAB function corr

% does not support gpuArrays, therefore when using the GPU, the

% correlation equation is hard-coded.

if useGPU

% correlation coded explicitly, as "corr" is not GPU supported yet

% remove means

PEseed = PEseed - mean(PEseed);

PEortho = bsxfun(@minus,PEortho,mean(PEortho,2));

% correlate

RorthoAB(:,iVertex) = sum(bsxfun(@times,PEortho,PEseed),2) ./ ((sum(PEortho.^2,2)).^.5 * (sum(PEseed.^2)).^.5);

else

RorthoAB(:,iVertex) = corr(PEseed',PEortho');

end

% Update status bar.

if mod(iVertex,10) == 0

waitbar(iVertex/size(SS,1),waitbarHandle,sprintf('Calculating orthogonalized connectivity.\nVertex %u of %u complete.',iVertex,size(SS,1)));

end

end

delete(waitbarHandle);

if useGPU

RorthoAB = gather(RorthoAB);

tol = gather(tol);

reset(gpu);

end

% 8. Calculate the symmetric, corrected orthogonalized connectivity matrix

% (Rortho). For region of interest intersection connectivity analyses, a

% symmetric connectivity matrix is required. Therefore, RorthoAB is averaged

% with its transpose. Due to underestimation of correlation inherent to

% orthogonalization, a correction factor of 0.578499 is applied. See Hipp,

% J.F. et al., 2012. Large-scale cortical correlation structure of spontaneous

% oscillatory activity. Nature Neuroscience, 15(6), pp.884–890. for the basis of

% this.

Rortho = ((RorthoAB + RorthoAB') ./ 2) ./ 0.578499;

% 9. Limit the correlation values of Rplain and Rortho to |1 - tol|. This is

% done to prevent non-finite values in the Fisher R to Z transform of the

% correlation coefficients (i.e., fisherz(1) = Inf).

Rplain(Rplain >= 1) = 1 - tol;

Rplain(Rplain <= -1) = -1 + tol;

Rortho(Rortho >= 1) = 1 - tol;

Rortho(Rortho <= -1) = -1 + tol;

end
